# Projection-based respiratory-resolved left ventricular volume measurements in patients using free-breathing double golden-angle 3D radial acquisition

**DOI:** 10.1007/s10334-018-0727-3

**Published:** 2018-12-12

**Authors:** Karen Holst, Alexander Fyrdahl, Kenneth Caidahl, Martin Ugander, Andreas Sigfridsson

**Affiliations:** 1 Department of Clinical Physiology, Karolinska Institutet and Karolinska University Hospital, Stockholm, Sweden; 2 Department of Molecular and Clinical Medicine, Institute of Medicine, Sahlgrenska Academy, Gothenburg University, Gothenburg, Sweden

**Keywords:** Cardiac imaging, Respiratory resolved, Golden-angle, Radial, Three-dimensional, Self-gating

## Abstract

**Objective:**

To refine a new technique to measure respiratory-resolved left ventricular end-diastolic volume (LVEDV) in mid-inspiration and mid-expiration using a respiratory self-gating technique and demonstrate clinical feasibility in patients.

**Materials and methods:**

Ten consecutive patients were imaged at 1.5 T during 10 min of free breathing using a 3D golden-angle radial trajectory. Two respiratory self-gating signals were extracted and compared: from the *k*-space center of all acquired spokes, and from a superior–inferior projection spoke repeated every 64 ms. Data were binned into end-diastole and two respiratory phases of 15% respiratory cycle duration in mid-inspiration and mid-expiration. LVED volume and septal–lateral diameter were measured from manual segmentation of the endocardial border.

**Results:**

Respiratory-induced variation in LVED size expressed as mid-inspiration relative to mid-expiration was, for volume, 1 ± 8% with *k*-space-based self-gating and 8 ± 2% with projection-based self-gating (*P *= 0.04), and for septal–lateral diameter, 2 ± 2% with *k*-space-based self-gating and 10 ± 1% with projection-based self-gating (*P *= 0.002).

**Discussion:**

Measuring respiratory variation in LVED size was possible in clinical patients with projection-based respiratory self-gating, and the measured respiratory variation was consistent with previous studies on healthy volunteers. Projection-based self-gating detected a higher variation in LVED volume and diameter during respiration, compared to *k*-space-based self-gating.

## Introduction

Cardiac magnetic resonance (CMR) imaging is a routine part of the diagnostic work-up in heart disease. An advantage of CMR is the variety of physiological and dynamic imaging methods available. However, many of the methods are still sensitive to respiratory motion, which is often resolved by performing the acquisition during breath holding. Breath holding can be difficult for patients to sustain and often leads to degraded image quality and motion blurring [1] or misalignment between stacks of two-dimensional (2D) image slices acquired during separate breath-holds [2, 3]. Furthermore, an intrinsic part of breath holding is that only one respiratory phase is imaged. As an alternative solution, free-breathing CMR techniques have been developed that allow acquisitions to be long enough for three-dimensional (3D) imaging. Cardiac imaging during free breathing also enables reconstruction of respiratory resolved images [4–6]. Bridging the gap between research in healthy individuals and clinical feasibility is an important step that often proves difficult. Patients have a higher variability in respiratory breathing patterns, heart rates, and ability to lie still during a long examination, thus making successful imaging challenging [7].

Patients who would benefit from respiratory-resolved evaluation of LV volume are those with stiffness changes in the myocardium or pericardium [8]. Filling of the right ventricle is affected by the pressure changes in the chest during respiration in healthy individuals [9]. When the pressure is high during expiration, the venous return to the right atrium becomes restricted, resulting in a shift of the septum to the right, thus allowing an increase in LV filling. During inspiration, the lower chest pressure increases the venous return to the right side of the heart and thereby shifts the septum to the left, which decreases the filling of the left ventricle. The left ventricular filling pattern during respiration in patients with stiffness changes can become either increased or decreased compared to normal [10, 11]. Distinguishing between diseases that present with similar symptoms, but have different etiologies and treatments, such as restrictive cardiomyopathy and constrictive pericarditis, could potentially be performed by measuring the respiratory variation in LV volumes.

Measurements of variation in LV volumes during respiration by 3D imaging has been attempted in healthy volunteers [12], but has to our knowledge never been tested in patients. Respiratory variation in LV function during free breathing has been studied in patients by measuring septal excursion with 2D imaging [10, 13]. However, free breathing 2D imaging is sensitive to through-plane motion and provides only a regional assessment of the respiratory-induced variation.

A method for respiratory-resolved free breathing 3D acquisition has previously been developed [12, 14], evaluated in healthy individuals, and successfully illustrated the respiratory variation in LV end-diastolic volumes (EDV) [14]. Two challenges were identified in the method; reduced image quality caused by eddy current effects, and sensitivity to changes in respiratory patterns with the respiratory self-gating method. The eddy current artifacts were caused by rapid movement in *k*-space from repetition time to repetition time when using the golden-angle trajectory. The respiratory self-gating did not take the amplitude of respiration into account, and could lead to situations where data, acquired in cycles with higher amplitudes or different shape, were sorted into the wrong respiratory phase bins. Furthermore, the *k*-space-based self-gating required a precise positioning of a band-pass filter, which might not be able to account for greatly varying frequencies in patients. In this work, the acquisition was modified to address these previously identified challenges. Specifically, the golden-angle trajectory was modified to decrease the mean angle between consecutive spokes from 92° of the original golden-angle trajectory to 7° to reduce image artifacts from eddy currents, and a new projection-based respiratory self-gating strategy was implemented. LVEDV and LVED diameters from both self-gating strategies were compared and tested in patients consecutively referred for clinical evaluation.

## Materials and methods

### Clinical cardiac patients

Data were acquired from ten patients (48 ± 16 years, 6 males) referred for a clinical CMR scan for cardiac evaluation. The study was approved by the regional human subject research ethics review board, and all patients provided written informed consent. Data acquisition for this study was added to the standard clinical protocol and was performed prior to administration of contrast agent.

### Data acquisition

Images were acquired using a clinical 1.5-T MRI scanner (MAGNETOM Aera, Siemens Healthcare, Erlangen, Germany) during free breathing and with a spine and a chest multi-channel surface coil with 30 active channels in total. The acquisition was undertaken using a free-running double golden-angle 3D radial trajectory [15] incorporated in a prototype-balanced steady-state free precession sequence. The double golden-angle trajectory was given by an azimuthal angle *α* and a polar angle *β* calculated from two golden means *ϕ*_1_ and *ϕ*_2_ as:

1 $$\begin{aligned} \alpha & = 2\pi \,\left\{ {m\phi_{2} } \right\} \\ \beta & = \cos^{ - 1} \left\{ {m\phi_{1} } \right\} , \\ \end{aligned}$$

where *m* was the spoke number and {} denoted the modulo one operation. Conventionally, the golden means used are *ϕ*_1_  = 0.4656 and *ϕ*_2_ = 0.6823. In this work, *ϕ*_1_  = 0.0219 and *ϕ*_2_ = 0.0102 were used to reduce the angle between consecutively acquired radial spokes from 92 ± 13° to 7 ± 8° (mean ± standard deviation) to reduce image degradation from eddy current-related artifacts [16]. A superior–inferior radial spoke for respiratory self-gating was incorporated into the sequence, and was acquired every 25th repetition time (every 64 ms) to detect the motion of the diaphragm during respiration. With the superior–inferior spoke, the corresponding mean angles are 90 ± 17° and 11 ± 17° for the original and the reduced angular step, respectively.

Imaging parameters were: matrix size 176 × 176 × 176, isotropic voxel size 2 × 2 × 2 mm^3^, repetition time/echo time 2.5/1.3 ms, flip angle 60°, 250,000 radial spokes, including 10,000 superior–inferior spokes for projection-based self-gating, which were not used for image reconstruction. The 3D image volume was manually positioned over the heart, but not angled, and total scan time was 10 min. ECG and bellows signals were simultaneously acquired.

Phantom imaging was performed in a bottle containing a nickel sulfate solution (NiSO_4_), once with the original golden-angle trajectory and once with the modified angle increment. Imaging parameters for the phantom experiment were: matrix size 176 × 176 × 176, isotropic voxels 1.4 × 1.4 × 1.4 mm, TR/TE 2.9/1.4 ms, flip angle 60°, 48,657 radial spokes, corresponding to a fully sampled radial *k*-space. Images were reconstructed from all data of the free-running acquisition. To evaluate the robustness to physiological binning, a healthy volunteer (age 25 years, male) was also imaged with both methods. The parameters used for this experiment were: matrix size 176 × 176 × 176, isotropic voxels 2 × 2 × 2 mm, TR/TE 2.5/1.3 ms, flip angle 60°, 250,000 radial spokes, of which 10,000 were superior–inferior self-navigation spokes.

### *k*-Space-based respiratory self-gating

The *k*-space-based respiratory self-gating has been described in detail previously [12, 14]. In short, the *k*-space-based self-gating signal was extracted from a matrix consisting of the *k*-space center sample in each spoke for all coil elements. First, a one-dimensional signal was found by combining all coil elements, and second, the signal was band-pass filtered to include only respiratory frequencies. The coils were combined using a reference vector consisting of the *k*-space center sample from one radial spoke for all coil elements. The one-dimensional signal was then obtained from the scalar product of the reference vector and all other vectors of center samples over coils. Respiratory phase was extracted from an analytical signal and the phase wraps of the analytical signal were used to distinguish between respiratory cycles.

Only respiratory phase information was contained in the *k*-space-based self-gating signal. The position of mid-inspiration was determined as 28% of the respiratory cycle relative to end-expiration, and mid-expiration as 88% of the respiratory cycle relative to end-expiration, corresponding to phase bins 1 and 6 in our previous work [14].

### Projection-based respiratory self-gating

For projection-based respiratory self-gating, the respiratory signals were extracted from the superior–inferior spokes, which were acquired with a frequency of 15.7 Hz. Projection-based respiratory self-gating has been described previously [17, 18] and the respiratory self-gating signals were extracted in five steps adapted from [19]. First, the projection in image space of all the superior–inferior spokes was calculated using a one-dimensional Fourier transformation along the spoke direction, providing spatial information about the structures along the direction perpendicular to the diaphragm over time. Second, principal component analysis was performed coil-wise for all projections. Third, locally weighted scatterplot smoothing was performed with a span of six points. Fourth, spectral clustering of the two dominant principal components from each coil element was performed to estimate a single respiratory self-gating signal. Fifth, slowly varying trends in the self-gating signal were removed with a second-, third- or fourth-order polynomial fit, depending on the degree of signal variation that was caused by other factors than the respiratory motion.

To divide individual respiratory cycles, an analytical signal of the projection-based respiratory self-gating signal was extracted using the Hilbert transform. Cycle starting points were extracted from the phase angle of the analytical signal at 2π wrapping points. A mid-inspiration and a mid-expiration point were determined within each respiratory cycle using the maximum amplitude of the cycle and the minimum amplitudes in the beginning and end of the cycle. The mid-inspiration point was positioned where the self-gating signal amplitude was exactly halfway between the minimum in the beginning of the cycle and the maximum of the cycle. The mid-expiration point was positioned at the amplitude half of the maximum of the cycle and the minimum at the end of the cycle.

### Data binning of respiratory pressure extremes

Each radial spoke was assigned a combination of respiratory and cardiac phase. Data from two bins representing end-diastole in mid-inspiration and mid-expiration, corresponding to the minimum and maximum intra-thoracic pressure, were reconstructed. The reconstructed diastolic window has a width of 12.5% and starts at 70% of the RR interval. The width of the respiratory bins was defined as 15% of the respiratory cycle duration and centered on the points of mid-inspiration and mid-expiration found from the self-gating signals, as identified using both the projection-based and the *k*-space-based methods.

### Image reconstruction

Images from the data bins were reconstructed using conjugate gradient SENSE [20] with four iterations. The number of iterations was chosen by visual inspection, considering the trade-off between improved image quality and noise amplification with increasing number of iterations. Adaptive calculation of coil sensitivity maps was done from the first 10,000 consecutive radial spokes in each acquisition as previously described [21].

For every reconstructed data bin, the image volume was rotated using multi-planar reformatting to obtain short-axis, 2-, 3- and 4-chamber views. To avoid degradation from the additional interpolations associated with multi-planar reformatting in the final image, the calculated angles were used to transform the *k*-space coordinates prior to the gridding step. Image slices of 8-mm thickness were calculated by summing four complex-valued 2-mm slices in each of the four cardiac views to limit the number of slices for segmentation and improve the signal-to-noise ratio.

### Left ventricular volume segmentation

Manual segmentation of the left ventricular endocardial border was performed using the freely available software Segment 2.1 R5874 (Medviso, Lund, Sweden) [22]. Segmentation was performed for each patient in mid-inspiration and mid-expiration in end-diastole, for both of the self-gating methods. All segmentations were performed by a single observer (KH), and were reviewed by an experienced physician.

### Respiratory-induced change in left ventricular diameter

Segmentation of the LV endocardial border was used to measure the LVED volumes and diameters. The diameter was measured in the septal–lateral direction and the anterior–inferior direction. One mid-ventricular short-axis image during mid-inspiration and mid-expiration from each patient was used for these measurements.

LV segmentation was used for automatic identification of the centroid point in each mid-ventricular image slice. An anterior–inferior line was thereafter manually angled to intersect the centroid and the anterior–inferior diameter was measured as the distance between the two points on the segmented endocardial border intersecting this line. The septal–lateral diameter was measured in the same way from a line perpendicular to the anterior–inferior direction while intersecting the centroid.

### Image quality assessment

The difference between the original and the modified angle increment was evaluated by calculating a normalized average absolute deviation (NAAD) of the signal intensity in the phantom images, defined as:

2 $${\text{NAAD}} = 1 - \frac{{\mathop \sum \nolimits_{i = 1}^{N} \left| {s_{i} - } \mu\right|}}{\mu N} ,$$

where *s* is the signal intensity in a voxel, *μ* is the mean within the volume and *N* is the number of voxels within the volume. To minimize noise contributions to the deviation, the images were convolved by a 9-point cosine filter kernel prior to the calculation. Only voxels with at least 25% of the maximum intensity were considered.

To evaluate potential differences in image quality between the two self-gating methods, two blinded observers were presented with one short-axis stack at a time and were asked to rate the image quality on a scale from 1 to 5, where 1 = non-diagnostic, 2 = poor, 3 = adequate, 4 = good and 5 = excellent. The image stacks were presented in a randomized order.

### Statistics

Continuous data are presented as mean ± standard error of the mean (SEM) and discrete data as mean ± standard deviation (SD). Volume difference between self-gating methods and observer scores was tested with the Wilcoxon signed rank test using MATLAB (version R2016b; Mathworks, Natick, MA, USA). Interobserver agreement was assessed using Spearman’s rank correlation coefficient (*ρ*). *P *< 0.05 was considered statistically significant.

## Results

Patient characteristics are presented in Table 1.

**Table 1:** Patient information

Patient	Age	Sex	CMR diagnosis	LVEDVI (ml/m^2^)	LVEF (%)
1	41	F	Cardiac sarcoidosis	68	53
2	28	F	Normal findings	92	64
3	30	M	Myocarditis (acute)	101	58
4	63	M	Dilated cardiomyopathy	163	34
5	54	M	Near-normal findings	112	55
6	50	M	Myocarditis (non-acute)	77	60
7	73	F	Myocarditis (non-acute)	68	60
8	59	M	Dilated cardiomyopathy	154	45
9	59	F	Normal findings	73	57
10	26	M	Normal findings	92	66

LVED volume index (LVEDVI) and LV ejection-fraction (LVEF) were findings from the clinical CMR exam

### Self-gating and binning

**Fig. 1:**
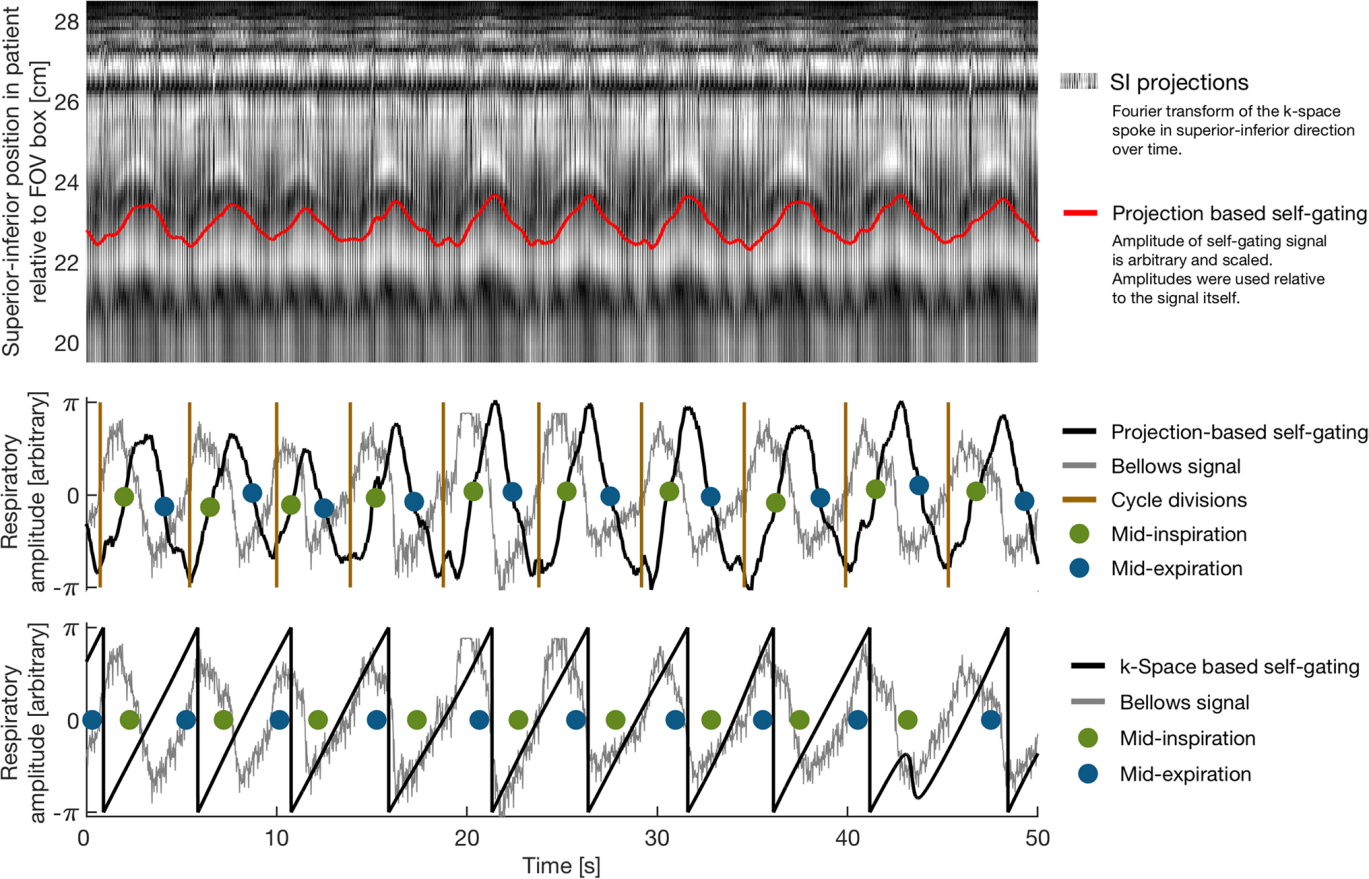
Examples of respiratory self-gating signals. Top and middle: Projection-based self-gating signal. Top: superior–inferior projections were used to detect the respiratory motion perpendicular to the diaphragm resulting in a projection-based self-gating signal (red). Middle: projection-based self-gating signal (black) compared to the measured bellows signal (gray). The analytic signal angle of the self-gating signal provided the respiratory cycle divisions shown as brown vertical lines, which represented end-expiration. Binning points for mid-inspiration (green) and mid-expiration (blue) were extracted in each cycle using the amplitude of the projection-based self-gating signal. Note how the peak excursion of the projection-based self-gating signal roughly follows that of the bellows signal, with a slight phase shift. Bottom: *k*-space-based respiratory self-gating signal (black) compared to the measured bellows signal (gray). The *k*-space-based self-gating signal only provided phase information about the respiration and the 2π-phase wrappings (vertical rapidly decreasing lines) were used to distinguish each respiratory cycle. Note how the amplitude of the *k*-space-based self-gating signal keeps the same amplitude and only respiratory phase information could be extracted. Also note the change of phase in the first three respiratory periods compared to the following respiratory periods and that the classification of mid-inspiration and mid-expiration is not consistent. 50 representative seconds of the total 10-min scan time are shown

Figure 1 shows an example of a projection-based respiratory self-gating signal compared to both the superior–inferior projections (top) and the measured bellows signal (middle). The amplitude of the self-gating signal is given in arbitrary units, proportional to the amplitude of the respiratory motion. Vertical lines show the division of individual cycles from the phase angle of the analytical signal. A constant phase shift was observed between the projection-based self-gating signal and the bellows signal in all the patients, as can be seen in Fig. 1.

Figure 1, furthermore, shows a *k*-space-based self-gating signal compared to the bellows signal (bottom). It demonstrates how the maximum and minimum amplitudes of each cycle were always ± π with no relation to the amplitude of the bellows signal. Only the phase of each respiratory cycle was considered, regardless of the shape and amplitude. The duration between the vertical phase wraps should follow the duration of the respiratory cycles seen in the bellows signal. However, as can be seen in Fig. 1, this was not always the case with *k*-space-based self-gating. This problem was observed for all the patients.

All curves in Fig. 1 show the same 50-s time period from a representative patient with the calculated mid-inspiration and mid-expiration points used for binning indicated by the green and blue circles. On average, 4199 ± 286 spokes were sorted into each bin for all patients, respiratory phases, and type of self-gating signals. This corresponds to a mean acceleration factor *R* = 11.6 with respect to radial Nyquist.

### Images

**Fig. 2:**
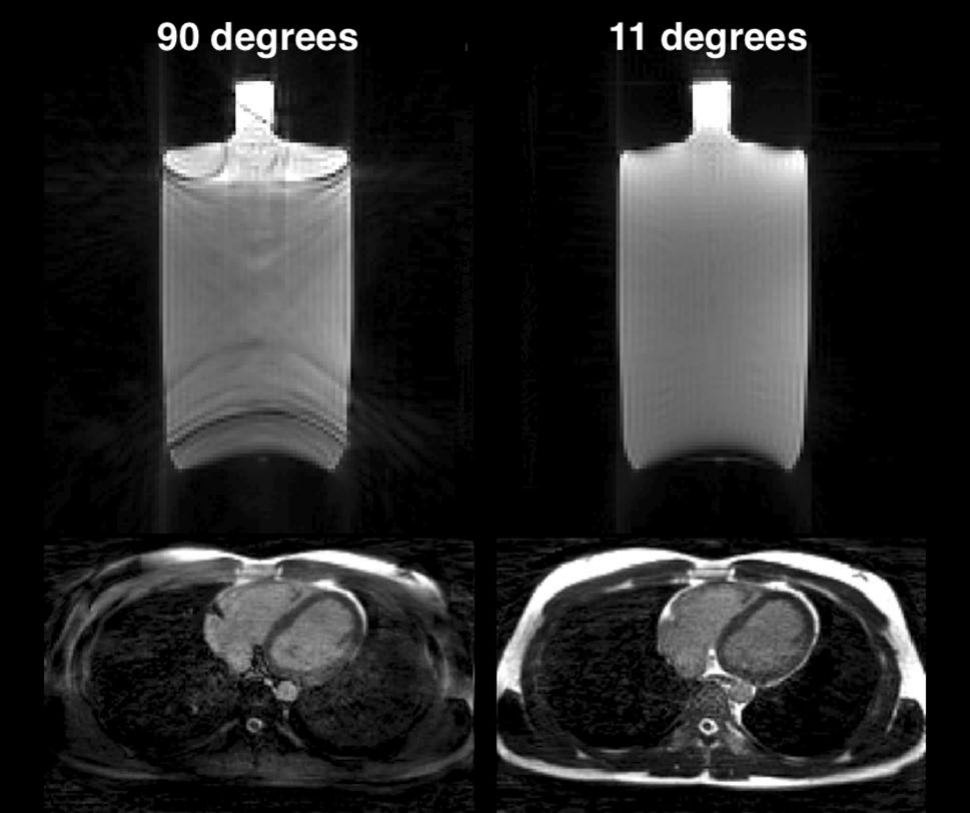
Phantom and in vivo experiments demonstrate effect of reducing the mean angle in the golden-angle radial trajectory. The left column corresponds to the original golden-angle trajectory with a mean angle of 90°, while the right column corresponds to the decreased mean angle of 11° which is used in this study, where the decreased mean angle trajectory exhibits less eddy current-induced image artifacts and a more homogenous fat signal across the image and less signal leakage in the lungs. Both the phantom experiment and the in vivo experiment were acquired with a superior–inferior self-navigation spoke which was removed prior to image reconstruction

**Fig. 3:**
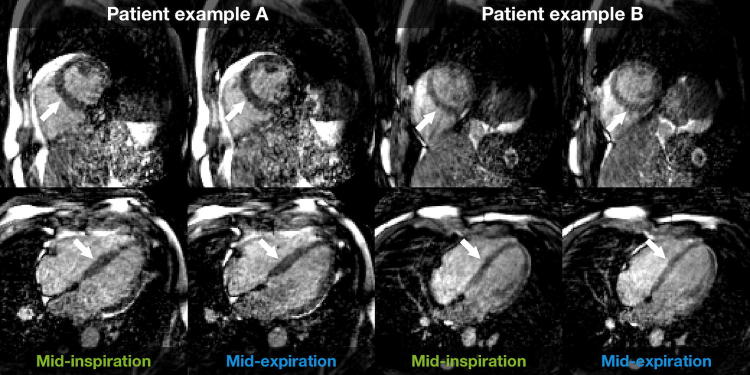
Representative images with projection-based respiratory self-gating from two patients. Images are mid-ventricular short-axis (top row) and four-chamber views (bottom row) from a full image volume covering the whole heart in end-diastole. Left column for each patient: mid-inspiration, representing minimum inspiratory pressure and thereby minimum LVEDV. Right column for each patient: mid-expiration, representing maximum expiratory pressure and thereby maximum LVEDV. Note how the septal position is placed further towards the right ventricle in mid-expiration and further towards the left ventricle in mid-inspiration making the LV larger in mid-expiration, indicated by the white arrows

**Fig. 4:**
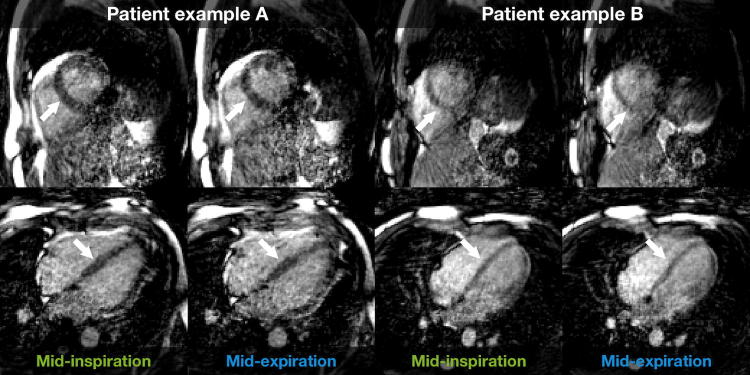
Representative images with *k*-space-based respiratory self-gating from two patients. Images are mid-ventricular short-axis (top row) and four-chamber views (bottom row) from a full image volume covering the whole heart in end-diastole. Left column for each patient: mid-inspiration, representing minimum inspiratory pressure and thereby minimum LVEDV. Right column for each patient: mid-expiration, representing maximum expiratory pressure and thereby maximum LVEDV. Using the *k*-space-based self-gating, it is more difficult to see the differences in septal position between the two reconstructed respiratory phases, indicated by the white arrows

**Fig. 5:**
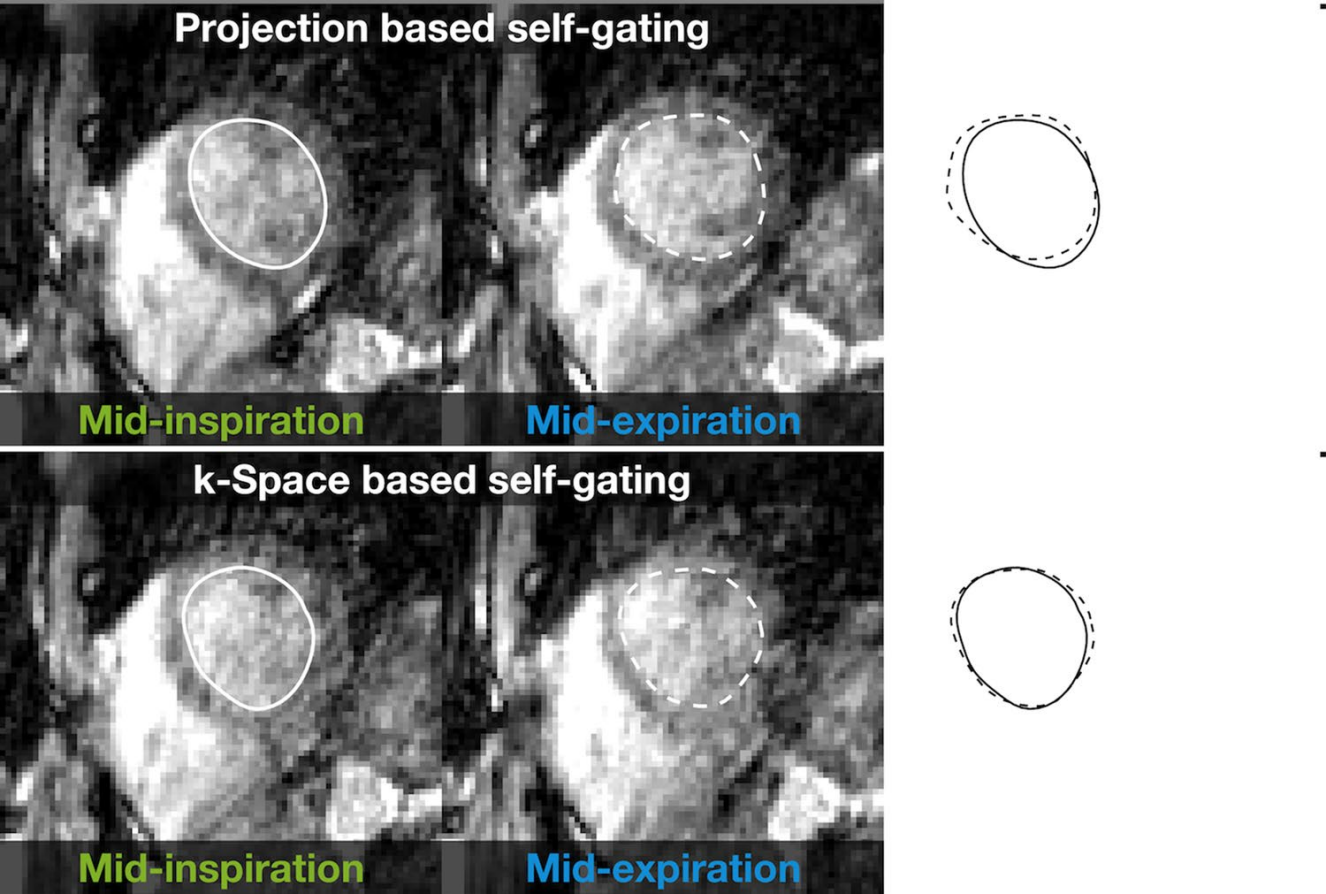
Mid-ventricular short-axis views from a representative patient. Top row: images with projection-based respiratory self-gating. Bottom row: images with *k*-space-based respiratory self-gating. Left: mid-inspiration corresponding to minimum inspiratory pressure and thereby minimum LVEDV. Center: Mid-expiration corresponding to maximum expiratory pressure and thereby maximum LVEDV. Right: dashed (maximum LVEDV) and solid (minimum LVEDV) endocardial segmentations are superimposed to illustrate the respiratory induced difference in ventricle size and shape, when reconstructed with the two different self-gating techniques

Results of the evaluation of the modified angle increment for the phantom experiment and the healthy volunteer are presented in Fig. 2. Representative examples of images in mid-inspiration and mid-expiration are shown in Fig. 3 for the projection-based self-gating and in Fig. 4 for the *k*-space-based self-gating. Figure 5 shows a mid-ventricular short-axis slice from one patient in both respiratory phases and with both respiratory self-gating methods. Note how the septal position is visibly shifted between mid-inspiration, representing minimum respiratory chest pressure and minimum LVEDV, and mid-expiration, representing maximum respiratory chest pressure and maximum LVEDV, with the projection-based self-gating. This septal shift is not visible in the images from the *k*-space-based self-gating. Furthermore, the septal myocardium-to-blood contrast appears to be higher with the projection-based self-gating than with the *k*-space-based self-gating, see Figs. 3, 4 and 5.

### Left ventricular volume measurements

**Fig. 6:**
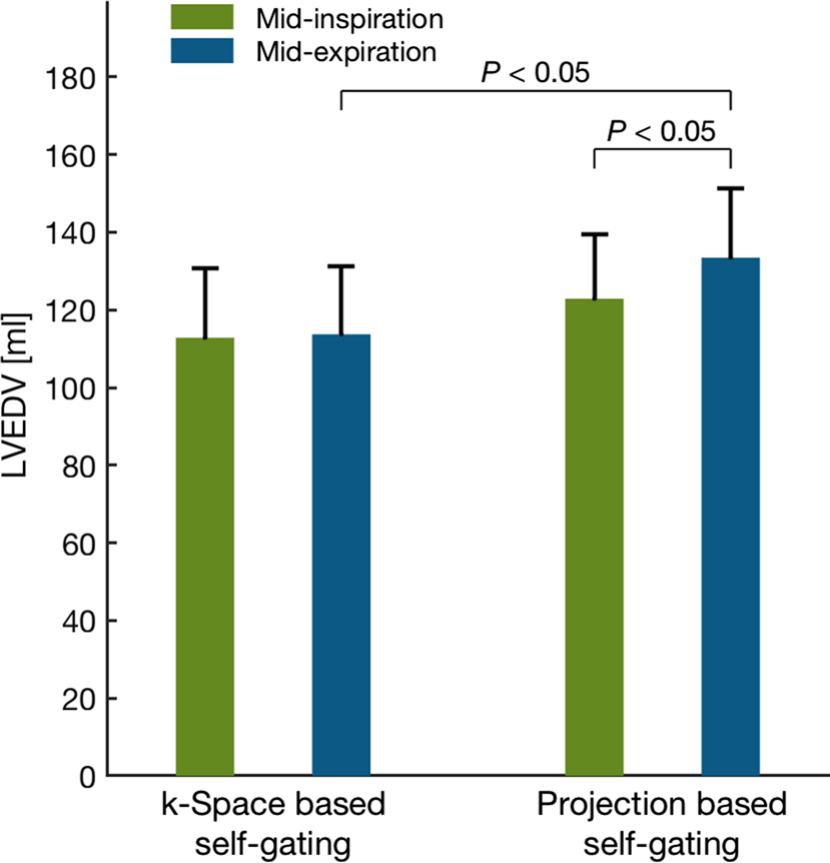
Mean left ventricular end-diastolic volume (LVEDV) during mid-inspiration (green) and mid-expiration (blue). Left: LVEDV from *k*-space-based self-gating. Right: LVEDV from projection-based self-gating. LVEDV with the projection-based self-gating was higher in mid-expiration compared to mid-inspiration while no difference was found in LVEDV between mid-inspiration and mid-expiration for the *k*-space-based self-gating. Error bars denote SEM

Average LVEDV in mid-inspiration and mid-expiration for both self-gating methods is shown in Fig. 6. Maximum LVEDV (occurred at maximum respiratory pressure during mid-expiration) was 133 ± 18 ml for the projection-based self-gating and 114 ± 18 ml for the *k*-space-based self-gating. Minimum LVEDV (occurred at minimum chest pressure during mid-inspiration) was 123 ± 17 ml for the projection-based self-gating and 113 ± 18 ml for the *k*-space-based self-gating. The difference between the mid-inspiration and mid-expiration with the projection-based self-gating was 11 ± 2 ml (*P *= 0.002) and with the *k*-space-based self-gating was 1 ± 2 ml (*P *= 0.6).

The difference in absolute volumes between the two self-gating methods was for mid-inspiration 10 ± 5 ml (*P *= 0.06) and for mid-expiration 20 ± 6 ml (*P *= 0.004).

**Fig. 7:**
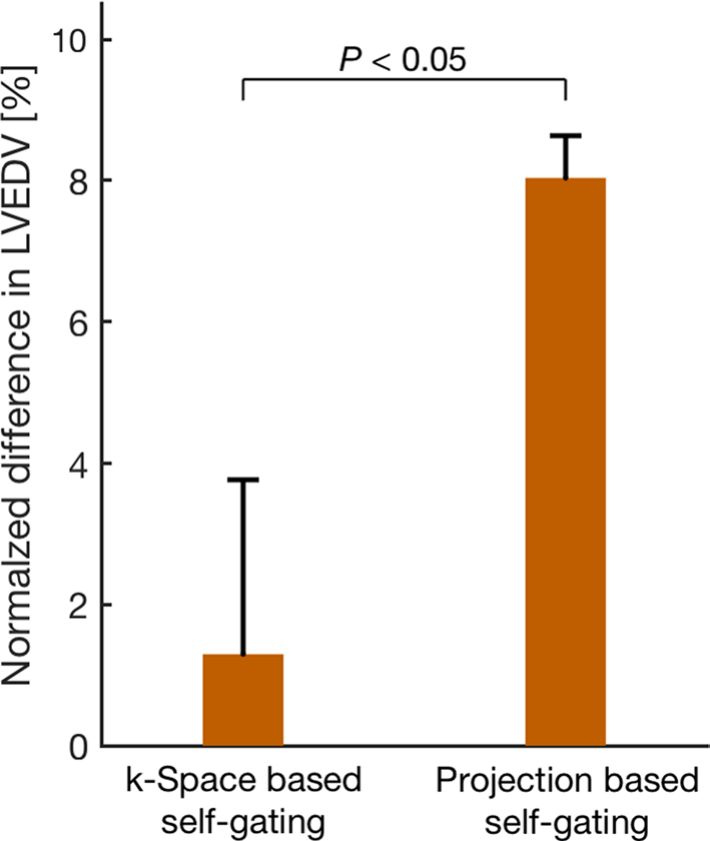
Mean difference between minimum LVEDV and maximum LVEDV relative to maximum LVEDV in percent for *k*-space-based respiratory self-gating (left) and projection-based respiratory self-gating (right). The difference in LVEDV was higher with the projection-based self-gating compared to the *k*-space self-gating. Error bars denote SEM

Respiratory-induced variation in LVEDV for each self-gating method is shown in Fig. 7. Average variation in LVEDV, expressed as difference between minimum and maximum LVEDV relative to maximum LVEDV, was 8 ± 2% for the projection-based self-gating and 1 ± 2% for the *k*-space-based self-gating (*P *= 0.04).

### Left ventricular diameter measurements

**Fig. 8:**
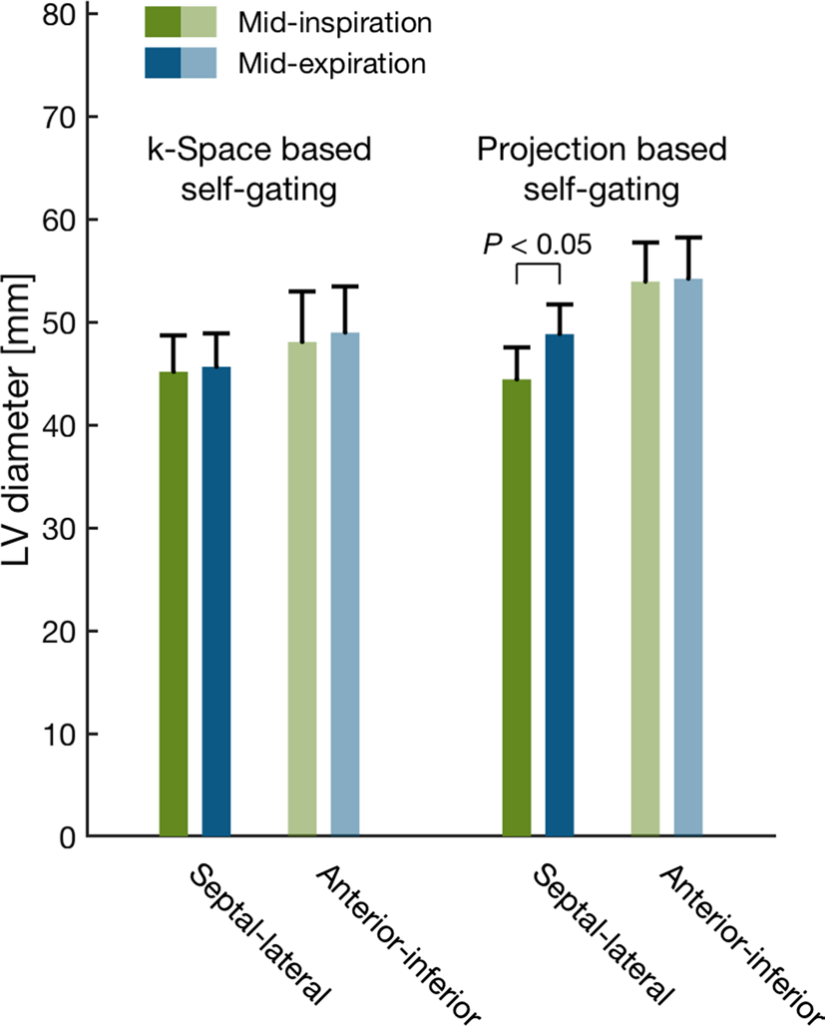
Mean LV diameter during mid-inspiration (green) and mid-expiration (blue). Left: LVEDV from *k*-space-based self-gating. Right: LVEDV from projection-based self-gating. LV diameters were measured in end-diastole from mid-ventricular short-axis images in septal–lateral direction (dark blue and green) and anterior–inferior direction (light blue and green). Only the septal–lateral diameter with the projection-based self-gating differed between mid-inspiration and mid-expiration. Error bars denote SEM

Average LVED diameters in mid-inspiration and mid-expiration for both self-gating methods are shown in Fig. 8. The difference in septal–lateral diameter between mid-inspiration and mid-expiration was 4.4 ± 0.5 mm (*P *= 0.002) with the projection-based self-gating and was 0.5 ± 0.6 mm (*P *= 0.7) with the *k*-space-based self-gating. The difference in anterior–inferior diameter between mid-inspiration and mid-expiration was 0.3 ± 0.8 mm (*P *= 0.9) with the projection-based self-gating and was 0.9 ± 1.0 mm (*P *= 0.6) with the *k*-space-based self-gating.

**Fig. 9:**
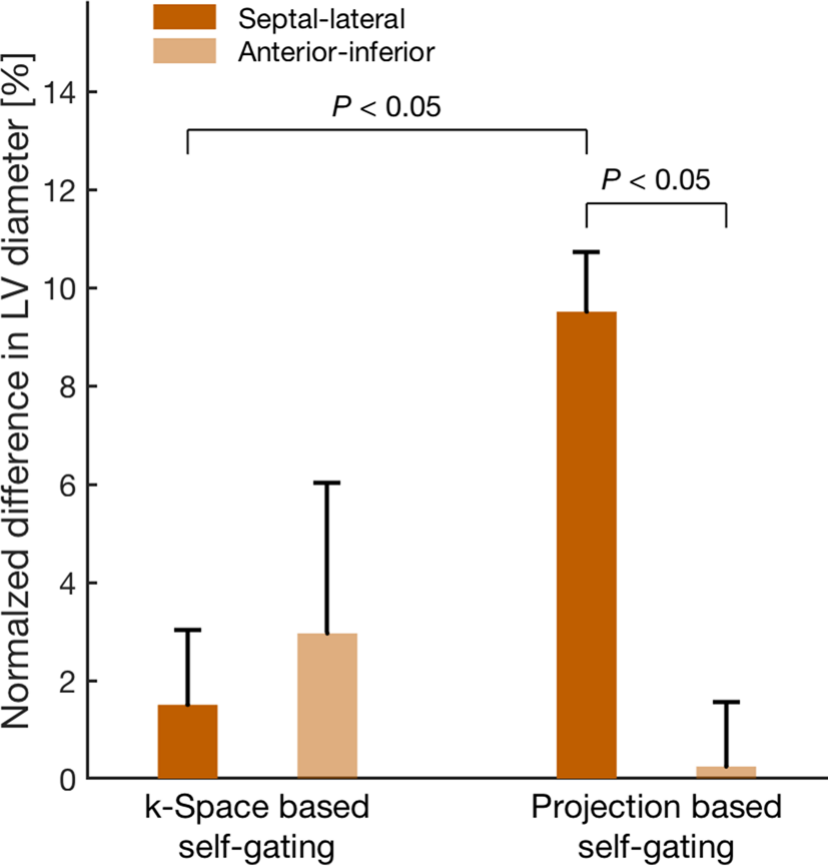
Mean respiratory-induced variation in LV diameter expressed as the difference in diameter between mid-inspiration and mid-expiration and normalized to mid-expiration. Variation in LV diameters in septal–lateral direction is shown in dark orange and anterior–inferior direction shown in light orange. Left: respiratory-induced variation in diameters measured in images from *k*-space-based respiratory self-gating. Right: respiratory-induced variation in diameters measured in images from projection-based respiratory self-gating. The variation in septal–lateral diameter from the projection-based self-gating was higher than from the *k*-space-based self-gating and no difference was found for the variation in anterior–inferior diameter between the two self-gating methods. Error bars denote SEM

Respiratory-induced variation in LV diameters for each self-gating method is shown in Fig. 9. The average variation in septal–lateral diameter, expressed as difference between minimum and maximum septal–lateral diameter relative to maximum septal–lateral diameter, was 10 ± 1% for the projection-based self-gating and 2 ± 2% for the *k*-space-based self-gating (*P *= 0.002). Average variation in anterior–inferior diameter, expressed as difference between minimum and maximum anterior–inferior diameter relative to maximum anterior–inferior diameter, was 0 ± 1% for the projection-based self-gating and 3 ± 3% for the *k*-space-based self-gating (*P *= 1.0).

### Image quality assessment

The NAAD was calculated to be 0.81 for the conventional angle increment and 0.85 for the modified angle increment, where a higher value corresponds to a more uniform image. The projection-based self-gating method obtained a higher score from the blinded observers compared to *k*-space center-based self-gating method (mean ± SD: 2.75 ± 0.77 vs. 2.11 ± 0.92, *P* = 0.005). Interobserver agreement was found to be *ρ* = 0.34 and the intraobserver agreement was estimated to be *ρ* = 0.54.

## Discussion

Golden-angle radial acquisitions in 3D and during free breathing with projection-based self-gating allows for measurement of respiratory-induced variation in LVEDV and LVED diameters in cardiac patients.

Phantom experiments suggest that eddy current-induced artifacts seem to be reduced using the smaller angular increment. Notably, the inclusion of a superior–inferior self-navigation spoke did not visibly impact the eddy current characteristics. An in vivo experiment in a healthy volunteer shows a more homogenous fat signal across the field of view.

Projection-based respiratory self-gating from superior–inferior spokes was able to detect the respiratory motion in terms of both respiratory phase and amplitude when visually comparing to the measured bellows signal. A phase shift between the bellows signal and the projection-based self-gating signal was present for all patients, likely caused by indirect coupling between the abdominal circumference and the diaphragmatic position during respiration. This illustrates the importance of an internal, and more direct, measurement of respiration other than respiratory bellows [23, 24]. Using amplitude detection within each respiratory cycle, adaptation to varying respiratory patterns is intrinsic to the method. In contrast, the *k*-space-based self-gating method relies on the analytic phase varying appropriately over the different cycles. The complex amplitude of the *k*-space center is not by necessity correlated with the amplitude of the motion, but rather a measure of the global signal changes over time. However, considering the spatial encoding provided by the surface coil elements, it might be possible to extract a self-gating signal related to the respiratory amplitude.

Analysis of the *k*-space-based respiratory self-gating signals shows that the *k*-space-based method is not consistently capturing the respiratory phase, which could explain why the *k*-space-based self-gating could not observe any respiratory-induced variation in LVEDV and LV septal–lateral diameters.

Images from representative patients show that the contrast between blood and myocardium was higher with the projection-based self-gating compared to the *k*-space-based self-gating. Especially the septal and anterior LV walls were more clearly defined with the projection-based method. Also, the heart–liver interface was more clearly defined with the projection-based self-gating. The interfaces were likely blurry when using *k*-space-based self-gating because the two respiratory phases were not identified correctly using that technique.

With the *k*-space-based self-gating, the blood–myocardial borders are poorly defined, especially in mid-expiration. Segmentation of the LV endocardial border indicates a larger difference in size, location and shape of the left ventricle, and the septal wall in particular, between mid-inspiration and mid-expiration with the projection-based self-gating compared to *k*-space-based self-gating. There is almost no difference in the segmentation with the *k*-space-based self-gating.

Absolute LVEDV measurements support the observations that projection-based self-gating was able to distinguish between mid-inspiration and mid-expiration and measured an 11-ml difference between the two phases, whereas the *k*-space-based self-gating was not able to detect any difference between the two phases. Also, the respiratory-induced variation in LVEDV was higher with the projection-based self-gating. The mid-expiratory LVEDV was higher with the projection-based self-gating compared to the *k*-space-based self-gating. This could be caused by the lower myocardium-to-blood contrast with the *k*-space-based self-gating making manual segmentation more difficult.

A previous study showed that the *k*-space-based self-gating was able to separate the mid-inspiration and mid-expiration in healthy volunteers and measured relative differences in mid-inspiratory LVEDV compared to mid-expiratory LVEDV of 5–6% [14]. In the current study, the *k*-space-based self-gating could not detect any difference between mid-inspiration and mid-expiration. Healthy volunteers likely have more stable breathing patterns in both amplitude and frequency than patients. The use of a fixed-frequency band-pass filter could potentially explain the bad performance in patients, if they had more varying respiratory frequencies. On the other hand, the difference in LVEDV between mid-inspiration and mid-expiration with the projection-based self-gating was 8%, which was even higher than measured in healthy individuals. This could potentially indicate that the *k*-space-based measurements in the healthy volunteers were indeed underestimations too, but less so than observed in patients. If the respiratory phases are not identified correctly, it is more probable that it leads to underestimation rather than overestimation of the volume or diameter differences. This has to be verified further by comparing the two respiratory self-gating methods in healthy volunteers before a conclusion can be made.

Measurements of LVED diameters corroborate the findings in volume measurements. Only the septal–lateral diameter measured from the projection-based self-gating detected a difference between mid-inspiration and mid-expiration. No respiratory-induced variation was found in anterior–inferior diameter with either of the self-gating methods indicating that the main contribution to the variation in LVEDV comes from the septal–lateral direction. This is supported by previous findings that the septum plays the main role in respiratory-induced variation in cardiac mechanics [9]. Septal excursion in healthy individuals has previously been found to be 6.6 ± 2.6 mm [10]. In the current study, the septal excursion was 4.4 ± 0.5 mm in patients with the projection-based self-gating.

A limitation of this study is that only two respiratory phases were reconstructed. Another limitation is the relatively small number of patients, which warrants future validation studies with power analysis based on the results in this initial study. Furthermore, the introduction of the self-gating projection spoke in the pulse sequence could potentially cause eddy current-induced oscillations, and it should be verified that this does not cause disturbing image artifacts. In the current implementation, the drift correction of the projection-based self-gating signal was performed manually in each patient, which is a limitation from a reproducibility standpoint. The images were reconstructed with the CG-SENSE method. With the imaging parameters used, the blinded observers deemed the image quality to be adequate to poor, on average. Potential improvement of image quality could be obtained by employing a more advanced reconstruction method such as Alternating Direction Method of Multipliers or Low-Rank and Sparse.

Projection-based respiratory self-gating enables 10-min acquisition of free-running 3D double golden-angle data in patients and is able to measure the variation in LVEDV and LV septal–lateral diameters between mid-inspiration and mid-expiration.

## Conclusions

Measuring respiratory variation in LV end-diastolic dimensions was possible in clinical patients with double golden-angle 3D imaging and projection-based respiratory self-gating. Projection-based self-gating from superior–inferior spokes detected a higher variation in LVEDV during respiration, compared to self-gating from *k*-space center. Respiratory-induced variation of LVEDV in patients with normal as well as reduced LVEF was 8% and respiratory-induced variation in end-diastolic septal–lateral mid-ventricular diameter was 10%. This respiratory variation was similar to earlier studies in healthy volunteers.
